# A Framework Phylogeny of the American Oak Clade Based on Sequenced RAD Data

**DOI:** 10.1371/journal.pone.0093975

**Published:** 2014-04-04

**Authors:** Andrew L. Hipp, Deren A. R. Eaton, Jeannine Cavender-Bares, Elisabeth Fitzek, Rick Nipper, Paul S. Manos

**Affiliations:** 1 The Morton Arboretum, Lisle, Illinois, United States of America; 2 The Field Museum, Department of Botany, Chicago, Illinois, United States of America; 3 University of Chicago, Committee on Evolutionary Biology, Chicago, Illinois, United States of America; 4 University of Minnesota, College of Biological Sciences, Saint Paul, Minnesota, United States of America; 5 Floragenex, Inc., Eugene, Oregon, United States of America; 6 Duke University, Department of Biology, Durham, North Carolina, United States of America; Montreal Botanical Garden, Canada

## Abstract

Previous phylogenetic studies in oaks (*Quercus*, Fagaceae) have failed to resolve the backbone topology of the genus with strong support. Here, we utilize next-generation sequencing of restriction-site associated DNA (RAD-Seq) to resolve a framework phylogeny of a predominantly American clade of oaks whose crown age is estimated at 23–33 million years old. Using a recently developed analytical pipeline for RAD-Seq phylogenetics, we created a concatenated matrix of 1.40 E06 aligned nucleotides, constituting 27,727 sequence clusters. RAD-Seq data were readily combined across runs, with no difference in phylogenetic placement between technical replicates, which overlapped by only 43–64% in locus coverage. 17% (4,715) of the loci we analyzed could be mapped with high confidence to one or more expressed sequence tags in NCBI Genbank. A concatenated matrix of the loci that BLAST to at least one EST sequence provides approximately half as many variable or parsimony-informative characters as equal-sized datasets from the non-EST loci. The EST-associated matrix is more complete (fewer missing loci) and has slightly lower homoplasy than non-EST subsampled matrices of the same size, but there is no difference in phylogenetic support or relative attribution of base substitutions to internal versus terminal branches of the phylogeny. We introduce a partitioned RAD visualization method (implemented in the R package RADami; http://cran.r-project.org/web/packages/RADami) to investigate the possibility that suboptimal topologies supported by large numbers of loci—due, for example, to reticulate evolution or lineage sorting—are masked by the globally optimal tree. We find no evidence for strongly-supported alternative topologies in our study, suggesting that the phylogeny we recover is a robust estimate of large-scale phylogenetic patterns in the American oak clade. Our study is one of the first to demonstrate the utility of RAD-Seq data for inferring phylogeny in a 23–33 million year-old clade.

## Introduction

For organisms in which ecological and morphological differences persist even in the face of interspecific gene flow [1], phylogeny estimation can be problematic [2]–[4]. This is a pronounced problem in many forest trees, in which interfertility, high rates of outcrossing, and large effective population sizes [5]–[7] make estimating phylogeny and patterns of trait evolution challenging. Oaks (*Quercus* L., Fagaceae) are notable for the difficulties they pose to systematists. Renowned as a “worst case scenario for the biological species concept” [8] due to apparent local interspecific gene flow [1], [9]–[18], widespread oak species nonetheless exhibit genetic coherence across broad geographic ranges [19]–[23].

Previous published studies utilizing chloroplast DNA (cpDNA) and low-copy nuclear gene (LCNG) data have recovered a provisional framework phylogeny for the genus *Quercus* and identified the relative position of *Quercus* within the Fagaceae [24]–[26]. This body of work identifies a predominantly American clade within *Quercus*, comprising sections *Quercus* (the white oaks sensu stricto, including the Virentes of the Americas and roburoids of Eurasia), *Lobatae* Loudon (the red or black oaks), and *Protobalanus* (Trelease) A.Camus (the intermediate or golden oaks). The fossil record sets the divergence of sections *Quercus* and *Lobatae* at a minimum of 23–33 mya [27], [28]. Additional molecular analyses using rDNA-ITS and 5S-IGS sequences also define this clade to the exclusion of the Cerris and Ilex clades of Eurasia [29]. Although the Eurasian white oaks of section *Quercus* are embedded in this predominantly American clade, we refer to it hereafter in the paper as the “American clade.” The monophyly of each section is strongly supported in all analyses.

More recent analysis using amplified fragment length polymorphisms (AFLPs) [30] has provided better understanding of relationships among and within sections *Quercus*, *Lobatae*, and *Protobalanus* of the American oak clade. However, while we have good reason to believe that model-based analysis of AFLPs should provide unbiased (though perhaps noisy) phylogenetic estimates [31]–[36], the difficulties of assessing fragment homology [37], [38] and accurately modeling the evolution of AFLPs in the absence of knowledge about the underlying sequence [35] limits their utility for phylogenetic inference. In the case of oaks, in which large numbers of loci seem to be necessary to accurately assess population history and species trees, a molecular marker is needed that samples large numbers of sequenced loci from across the genome.

Restriction-site associated DNA (RAD) comprises fragments of DNA that lie adjacent to all copies of a particular restriction enzyme recognition sequence in an individual's genome [39]–[42]. These fragments subsample from an individual's genome, enabling efficient generation of large numbers of genetic markers for a sample of individuals using massively parallel sequencing methods. The method provides a useful tool for surveying the genome of organisms like oaks, in which we need to sample broadly across the genome without the benefit of a sequenced reference genome. Most applications of RAD sequencing have been applied within species [40], [43]–[46] or among closely related species [47], [48]. RAD sequencing has been demonstrated to be feasible in principle to clades as old as 40–60 million years, using 50-bp sequence reads [49]. However, this estimate was based on simulated RAD sequencing of *Drosophila* genomes, and we are not aware of any studies that investigate the utility of RAD sequencing in estimating phylogenies from bona fide sequence data in a clade that spans this phylogenetic depth.

In this paper, we present a framework phylogeny of oaks using sequenced RAD data, focusing on the American oak clade. We use a recently developed pipeline for phylogenetic analysis of sequenced RAD data [50] to generate sequence matrices under alternative sequence clustering thresholds and analysis parameters and investigate combinability of data across sequencing runs using a replicated subset of individuals. We investigate possible alternative topologies captured within the concatenated dataset using a partitioned RAD approach that identifies suites of loci favoring the optimal and near-optimal trees. We then estimate the function and cellular localization of genes used in our phylogeny based on inferred homology of RAD loci to expressed sequences and assess the relative phylogenetic signal between inferred loci that match with high certainty to expressed sequence tags (ESTs) and those that do not to evaluate whether coding genes differ in phylogenetic signal from the dataset as a whole. Finally, we present a strongly resolved molecular phylogeny for relationships among the major clades of the American oaks and preliminary findings regarding biogeography of the white oaks.

## Methods

### Sampling

The target of this study is the predominantly American oak clade, comprising *Quercus* sections *Quercus*, *Lobatae*, and *Protobalanus*. We selected twenty species from this clade and one member of section *Cerris* Dumort. to serve as an outgroup, based on previous work in the genus [24], [25]. Acorns collected by members of the International Oak Society (I.O.S.) as part of their 2006 seed exchange or by JCB were grown in experimental greenhouses at University of Minnesota to provide live material for RAD sequencing. Herbarium specimens were prepared from seedlings that reached sufficient size (deposited at The Morton Arboretum herbarium [MOR]). Growth of new leaves was stimulated by moving plants from a coolhouse to a warm greenhouse. Leaves were covered in foil for 48–72 hours prior to removal from the plants to reduce plastid contribution to the final extraction. Two samples in this study (*Quercus sagraena* CUVN10 [51] and *Quercus virginiana* FLBA140 [22]) were collected from wild plants in the field and stored as leaf tissue at −80°C until extraction. *Quercus virginiana* was collected at San Felasco Hammock State Preserve, authorized by the Florida Division of Environmental Protection, Division of Recreation and Parks and approved by Clif Maxwell, District Park Biologist. The permit is good for any Florida oak species and covers Floridian seed collections maintained in the UMN greenhouse (e.g., *Q. michauxii*, Q. *nigra*, *Q. hemisphaerica*, *Q. lyrata*). Collection of *Q. sagraena* in Cuba in Pinar del Rio was conducted with permission of Dr. Antonio Lopez Almirall at the Museo Nacional de Historia Natural, La Habana, Cuba. None of the other field collections of oak species involved endangered or threatened species, and collection of acorns did not require written permits, as they were acquired from roadside or cultivated populations in unregulated areas.

### DNA extraction and RAD library preparation

DNA was extracted from fresh or frozen material using the DNeasy plant extraction protocol (DNeasy, Qiagen, Valencia, CA), with modifications that we have used for previous studies in oaks [20], [30]. DNA extractions were gel-quantified in agarose by visual comparison with the New England Biolabs 100 bp DNA Ladder (NEB, Ipswich, MA). Extraction concentrations ranged from 5–10 ng DNA/μl extraction. RAD sequencing library preparation was conducted at Floragenex following the methods of Baird et al. [39]. Initial library preparations using *Sbf*I (an 8-base cutter: 5′ — CCTGCA|GG — 3′; 3′ — GG|ACGTCC — 5′) were not successful, failing either at the sonication step (no sonication) or at the final PCR. A second trial with *Pst*I (a 6-base cutter: 5′ — CTGCA|G — 3′; 3′ — G|ACGTC — 5′) resulted in successful library preparations for all individuals except for two not reported on in this study. Assuming a GC-content of 40%, genome size of 500 Mb (both of which are typical of oaks [26]), and completely random draw of nucleotides, we expect about 72,000 *Pst*I cut sites in the oak genome. There was no obvious correlation between sequence quality and initial DNA concentration or material type (fresh vs. frozen).

### Illumina sequencing

RAD libraries were barcoded by individual and multiplexed on an Illumina/Solexa Genome Analyzer IIx as part of three separate sequencing runs, one in 2010, one in 2011, and one in 2012. The 2010 sequencing reads were 60 bp in length, including the 5-bp barcode and 5 bases of the *Pst*I recognition sequence (underlined: 5′ — CTGCAG — 3′). The 2011 and 2012 sequencing reads were 95 bp in length, after removal of the multiplex index sequences, but including the barcode and recognition sequence. To ease the comparison between 2010 and 2012 sequencing runs, the 2012 sequences were cut to 50 bp for this paper. Processed data were returned in the Illumina 1.3+ variant of the FASTQ format [52], with Phred quality scores for all bases [53]. Quality, read lengths, and base composition of FASTQ data were assessed in R v. 2.15.2 [54] using the ShortRead package [55].

### Data analysis I: Clustering

Data were analyzed following a custom pipeline that approximately follows the method of Catchen et al. [56]. The method is detailed in Eaton and Ree [50] and implemented in pyRAD (code.google.com/p/pyrad/; www.dereneaton.com/software). In brief, sequences are clustered first by individual, and highly similar sequences are clustered into “stacks.” In pyRAD, these stacks are generated using USEARCH [57], which allows sequences within clusters to vary in indels, nucleotide polymorphisms, and sequencing strand (direction). This is a departure from the “off-by-N” approach implemented in the popular STACKS software [56], in which stacks are composed of sequences that differ by no more than a threshold number of single nucleotide polymorphisms. After clustering, rates of heterozygosity and sequencing error are jointly estimated from the base counts observed across all sequences and sites and clusters using the likelihood equation of Lynch [58], and heterozygotes are inferred by a binomial probability based on these parameters. Bases that cannot be assigned with ≥95% probability are treated as unknown (*N*). Each resulting stack is referred to hereafter as a locus. As triploids and tetraploids are believed to be uncommon in oaks [26], [59], [60], any locus possessing more than 2 haplotypes within individuals after correcting for sequencing errors was discarded, under the assumption that it was composed in part of paralogous sequences (rather than only homologous sequences). For each individual, each locus is summarized into a consensus sequence, and these consensus sequences are then clustered among individuals to generate a data matrix for each locus. Because not every individual has a sequence for every locus, due to both sequencing coverage and mutation of the restriction site defining RAD loci, the resulting data matrix is expected to be incomplete.

Clustering for this study was conducted over a range of parameter values, as follows, with settings that we varied in this study indicated in square brackets:

- *Data quality of input sequences.* All nucleotides with Phred quality scores <20 were replaced with N's (base unknown), and reads with >5% N's were removed from analysis.
- *Percent similarity required to cluster sequences into a stack:* 0.88.
- *Minimum stack depth for each individual:* 6.
- *Percent similarity required to cluster individuals into a locus:* 0.88.
- *Minimum number of individuals per locus cluster:* analyses were conducted with a minimum of four individuals or ten individuals [m4, m10 respectively].
- *Maximum number of heterozygotes per locus within individuals:* 3 nucleotide positions.
- *Maximum number of heterozygotes per nucleotide position among individuals:* 2 individuals, under the assumption that including loci within which a given nucleotide position is heterozygous for more than two individuals out of the 20 sampled risks including paralogs in analysis.
- *Maximum number of variable sites within a locus:* 10.
- *Replicates from 2012 included in analysis:* yes [wRE] or no [noRE].

Inclusion of technical replicates and number of individuals required to constitute a locus had no effect at all on the topology of phylogenetic results. Consequently, all results presented in this paper represent the analysis with replicates included and a minimum of 10 individuals required per locus (‘m10wRE’) unless otherwise indicated.

To assess gross patterns of locus-sharing among individuals, pairwise Jaccard's distances [61] were calculated from a locus presence-absence matrix (where 1 indicates the presence of a locus, and 0 the absence) for the m10wRE clustering results. Under the Jaccard distance, the distance between individuals is a function of the percentage of loci recorded for both of them, normalized by the total number of loci scored for both of them. Thus, loci that both individuals lack should not bias the estimate of genetic distance between them, but comparisons of individuals that differ in sequencing coverage have a greater expected pairwise genetic distance due to a greater probability of missing loci in just one individual relative to pairs of individuals that have equal sequencing coverage. At the same time, mutations in restriction sites are expected to imprint phylogenetic history on the pairwise distance matrix based on locus presence and absence [31], [34], [62], across phylogenetic depths at which mutation continues to be an information-preserving process [63]. Thus locus-sharing and pairwise Jaccard's distance reflect both genetic similarity and locus-sampling error due to less than exhaustive sequencing. Pairwise distances were visualized using nonmetric multidimensional scaling in the vegan v. 2.0-5 package [64] of R v. 2.15.2 [54]. Effect of dimensionality on the ordination was assessed by performing a set of ordinations setting *K* = 1 to 10 axes, allowing a maximum of 50 replicate runs from random starting configurations for each ordination, then plotting final stress against dimensions. The *K* = 1 to 3 solutions exhibited reasonably large decreases in final stress (0.2809, 0.1580, and 0.1128 respectively). The *K* = 2 and *K* = 3 ordinations were rerun with a maximum of 2000 replicates from random starting configurations, and both converged on a best solution. As ordination is used descriptively here, as in most studies [65], only the *K* = 2 ordination is reported in this study. Sequences of *Q. michauxii* and *Q. acutissima* were excluded from ordinations, because low overlap in locus coverage between both of these species and the others analyzed in this dataset dominated the ordinations in preliminary analyses (not shown).

Clustered data [d6m4 and d6m10], a Phylip-style dataset for phylogenetic analysis, and R scripts for conducting analyses are archived in the Dryad Digital Repository (http://dx.doi.org/10.5061/dryad.ts2hj).

### Data analysis II: Phylogenetic analysis

To assess phylogenetic relationships, we used maximum likelihood as implemented in RAxML v7.2.6 [66]. Analyses were conducted using the GTRGAMMA general time reversible model of nucleotide evolution, with branch support assessed using 200 nonparametric bootstrap replicates. Analysis was conducted initially on two datasets: loci clustered with a minimum of 4 individuals per locus (m4 dataset), and loci clustered with a minimum of 10 individuals per locus (m10 dataset). Technical replicates were included in all analyses presented, but their exclusion did not affect phylogenetic results (trial analyses not shown). The m10 dataset is reported on throughout this paper except where indicated, and m10 results vary only insignificantly from m4 results.

### Data analysis III: Partitioned RAD analyses

We tested whether there exist cliques of loci supporting globally suboptimal trees using a new partitioned RAD phylogenetic analysis approach. This method is motivated by LeQuesne's [67] idea of identifying the largest suite of characters that support a single topology. In the partitioned RAD analysis presented here, we use this rationale to visualize how many loci support the optimal tree relative to neighboring, suboptimal trees rather than using these suites of loci to search for the best-supported tree, but the tools developed could easily be adapted to reverse successive weighting [68]. In the method presented, we first (1) *generate a candidate pool of trees for comparison* by pruning our tree to only unique ingroup species and generating 200 unique suboptimal trees using nearest-neighbor interchange (NNI). As there are only 34 unique 1-step NNI trees for our 20-taxon tree, we included 166 unique 2-step NNI trees in our pool of permuted suboptimal trees. This left us with a total of 201 trees to analyze for each locus. We then (2) *generate a set of unique trees for each locus*, by pruning the 201 trees to only those tips present in each locus. Because pruning renders the 201 trees no longer unique, we filter out non-unique trees and save an index telling which original tree corresponds to each of the new trees. At this step, we also eliminate loci that have fewer than four individuals and that do not have any potentially parsimony-informative characters. All locus-tree sets are exported for analysis, along with shell scripts for batch phylogenetic analysis. We then (3) *estimate for each locus-tree set the likelihood of each tree*. Site likelihoods are calculated in RAxML under the GTRGAMMA model. Finally, we (4) *plot the likelihood of each tree, calculated using the original data matrix, against the number of loci supporting each tree or disfavoring each tree.* At this point, for clarity, the candidate pool of loci is restricted to loci containing a minimum number of unique trees and a minimum span in log-likelihood, and a log-likelihood window is defined for identifying trees as supported vs. disfavored by each locus. When trees derive from a single distribution, we expect a linear relationship between the number of loci favoring a tree and the log-likelihood for that tree. Points above/to the left of a regression line have more loci favoring the tree than expected; points below/to the right of a regression line have fewer loci favoring the tree than expected. Prediction intervals can be used to identify outlier trees that are more strongly supported (in terms of number of loci) than expected for their likelihood. Because outliers may lie anywhere along the regression line, outlier effects on the regression slope (e.g., Cook's distance [69]) are not appropriate to identifying outliers. For analyses presented here, we treat each tree as an independent data point.

While this sort of data exploration is not a substitute for formal species-tree estimation methods [70], [71], [72], it complements them by providing a way of exploring tree space for alternative topologies that may be strongly supported by the data but obscured by the dominant signal in the data. As our sampling here is deliberately skeletal, ignoring much of the fine structure of the oak tree of life, we leave a fuller exploration of alternative phylogenetic methodologies in oaks to future studies with finer-scale sampling. Preliminary analyses were conducted over loci filtered to have a minimum of anywhere from 4 to 150 trees; log-likelihood range of 0.0 to 5.0; and likelihood thresholds of 0.5 to 2.0. Results were qualitatively the same across all preliminary analyses, and only the minimum of 20 trees, log-likelihood range of 4.0, likelihood threshold of 2.0 analysis is presented here. A 95% prediction interval is utilized to identify outlier trees. All analyses were conducted in RADami version 1.0-3 [73].

### Data analysis IV: Estimating homology to coding regions

Homology to coding regions for inferred loci was estimated by using local BLASTN [74] of consensus sequences from inferred loci in the m10 dataset to three datasets of expressed sequences: (1) the NCBI EST-others database, release 193.0; (2) the NCBI RefSeq RNA database release 56 (ftp://ftp.ncbi.nlm.nih.gov/blast/db); and (3) a database of ESTs from *Quercus robur* (ftp://ftp.ncbi.nih.gov/repository/UniGene/Quercus_robur/, last modified 1/11/2011). A threshold E-value of 9E–15 was used as the cutoff for considering consensus sequences homologous to the target sequences. A consensus sequence was generated for each locus across individuals using the consensusString function in Biostrings version 2.26.2 [75] of Bioconductor [76].

To investigate the Gene Ontology (GO) of loci, target sequences from the BLASTN searches were uploaded to BLAST2GO [77]. BLASTX against the dataset of plant/*Arabidopsis thaliana* protein sequences integrated into BLAST2GO was performed to determine if a target sequence codes for a protein. Annotation of GO terms associated with each BLASTX hit was performed by mapping and annotation steps in BLAST2GO using default settings. GO terms were exported as text files and combined with BLASTN results in Microsoft Excel, where the target sequence id served as reference to assign GO terms to all RAD consensus loci that BLASTed to at least one EST. To determine how many loci represent a particular GO term, a count was made against the total of all GO terms. This total was done once with all sequences that BLASTN to ESTs at E-values < 9E–15, and once with only sequences that match one and only one unique gene description.

Phylogenetic utility of loci inferred to be homologous to any of the coding regions screened was assessed by comparing consistency index, percent of nucleotides potentially parsimony informative, and mean bootstrap over all branches for the optimal tree. Apportioning of mutations at deeper vs. more distal branches was quantified as the ratio of ML-optimized branch lengths for the basalmost three splits on the tree to ML-optimized lengths of the branches subtending the terminal splits between technical replicates. Significance of these statistics was assessed by comparison to 100 replicate datasets subsampled at random from all loci in the dataset that did not BLAST with any success to the three EST databases, where the sample size (number of loci) in each replicate dataset is the same as the number of loci that we inferred to be homologous to at least one EST. The two-tailed p-value for each statistic estimates the Type-I error rate under the null hypothesis that loci estimated to be homologous to expressed sequences are phylogenetically indistinguishable from loci that are not homologous to any expressed sequences in the databases we surveyed. RogueNaRok [78], [79] was utilized to identify taxa that are phylogenetically unstable in the smaller datasets.

## Results

### RAD sequences

**Figure 1:**
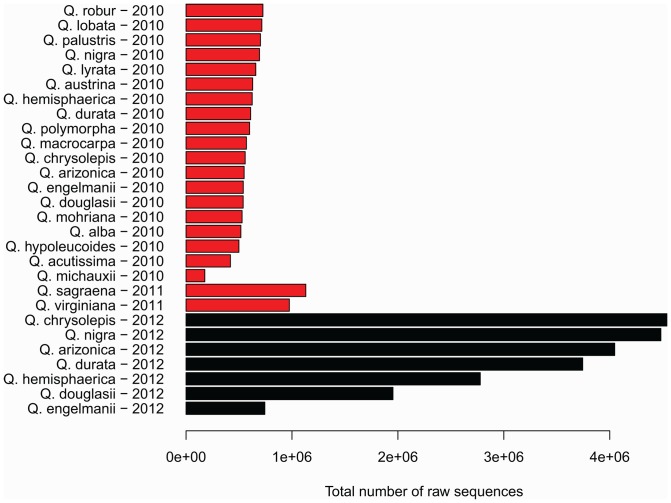
Number of sequences per individual, 2010, 2011, and 2012. Each sequencing run yielded between 1.77E5 and 4.54E6 sequences per individual. Sequences replicated in 2012 are shown in black.

For the year 2010 (initial) run, individuals yielded 177,168 to 725,871 sequenced reads (mean  = 558,006, sd  = 136,157) of 60 bp each (Fig. 1). For the 2012 (replicate) run, each individual yielded between 743,556 and 4,539,385 sequences (mean  = 3,056,861, sd  = 1,369,094) of 95 bp each (Fig. 1). This is a 5.5-fold increase in number of sequences yielded between 2010 and 2012. After removing the 5-bp recognition site and 5-bp barcode from each sequence, and ignoring decreases in quality toward the ends of the reads, this is a 15.4-fold increase in total sequence data per individual between 2010 and 2012. For this study, raw sequences from 2012 were truncated to a final length of 50 base pairs (excluding the barcode and *Pst*I recognition site) prior to clustering, so that read length differences between 2010 and 2012 should not bias similarity measures between individuals or phylogenetic results.

### DNA data matrix

Within individuals, the average number of sequences used to estimate the consensus sequence for each locus was 7.18±1.86 (standard deviation) for 2010 data, 29.19±11.2 for 2012 data. For the clustering results with replicates included, a total of 27,727 loci were inferred with a minimum of 10 individuals per locus (m10). Each locus includes an average of 16.3 individuals, making a 58.2% complete data matrix of 1,397,722 aligned nucleotides, of which 112,565 are variable and 51,378 are potentially parsimony-informative. For the m4wRE dataset, a total of 63,547 loci were inferred with a minimum of 4 individuals per loci; each locus includes an average of 10.5 individuals, making a 37.5% complete data matrix of 3,195,272 aligned nucleotides, of which 211,393 are variable and 84,599 are potentially parsimony-informative. Aligned loci, including gaps inserted in the course of alignment, range from 50 to 99 base pairs in length (m10 mean  = 50.41 bp; m4 mean  = 50.28 bp). Of the aligned bases, 427 (m10) to 1054 (m4) contained only Ns and were excluded from analysis. By comparison, the longest DNA-based dataset utilized in previous oak phylogenetic studies [30] utilized 2,932 AFLP bands, each of which reflects the evolution of 16 to 18 base pairs constituting the recognition sites flanking that band, a total of ca. 47,000 bp. Pairs of technical replicates share only 43–64% of the loci found in the union set of loci for the pair (Table 1). Locus coverage in the 2012 sequencing runs was 1.03- to 2.33-fold greater than the 2010 sequencing runs for the same individuals. 56.3% to 94.5% of loci found in the 2010 samples were also found in the 2012 samples (m10 mean  = 88.5%, m4 mean  = 85.2%).

**Table 1:** Loci recovered in 2010, 2012.

	2010 loci	2012 loci	Increase	Shared loci	Total loci	Overlap
***Q. arizonica***	15,321	24,428	59.4%	14,384	25,365	56.7%
***Q. chrysolepis***	13,325	22,797	71.1%	12,362	23,760	52.0%
***Q. douglasii***	14,082	22,860	62.3%	12,657	24,285	52.1%
***Q. durata***	15,296	24,351	59.2%	14,325	25,322	56.6%
***Q. engelmanii***	14,196	14,679	3.4%	8,653	20,222	42.8%
***Q.*** ** ***hemisphaerica***	14,390	20,718	44.0%	13,461	21,647	62.2%
***Q. nigra***	14,905	21,090	41.5%	14,079	21,916	64.2%
**Average**	14,502	21,560	48.7%	12,846	23,217	55.2%

RAD libraries were prepared once for each of the seven individuals shown and sequenced separately on an Illumina sequencing platform for each analysis to create technical replicates. *Column headings:* The number of loci recovered per individual (“2010 loci,” “2012 loci”) is based on the clustering pipeline described in methods. “Increase” is the percent increase in locus number by individual from 2010 to 2012. “Total loci” is the total number of loci that recovered from either the 2010 or 2012 sequencing run for each individual (the union set). “Shared loci” is the total number loci recovered in both the 2010 and the 2012 sequencing run for each individual (the intersection set). “Overlap” is shared loci expressed as a percentage of total loci.

### Phylogeny

**Figure 2:**
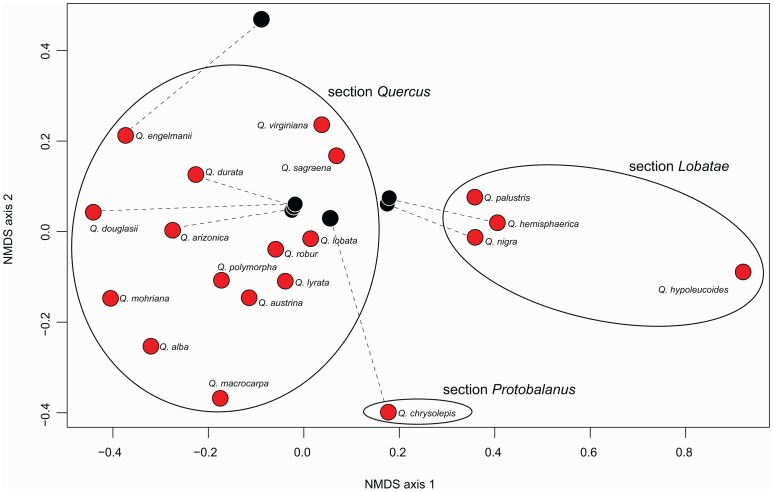
Phylogenetic tree of *Quercus* based on maximum likelihood analysis of sequenced RAD data. Branch lengths are scaled in substitutions per nucleotide (scale bar in lower left). All individuals are from the 2010 sequencing run, except replicates labeled “2012.” Bars on the right side of the figure indicates the four *Quercus* sections sampled.

Analysis of the aligned data matrix recovers section *Lobatae* as sister to sections *Quercus* and *Protobalanus*, and all three of these as monophyletic insofar as we have sampled them (Fig. 2). It also places the live oaks of the *Virentes* group sister to the remainder of section *Quercus*. All of these relationships are recovered with 100% bootstrap support. This topology has also been recovered in previous phylogenetic studies on oaks based on DNA sequences [24] and AFLP data [30], but with lower statistical support.

**Figure 3:**
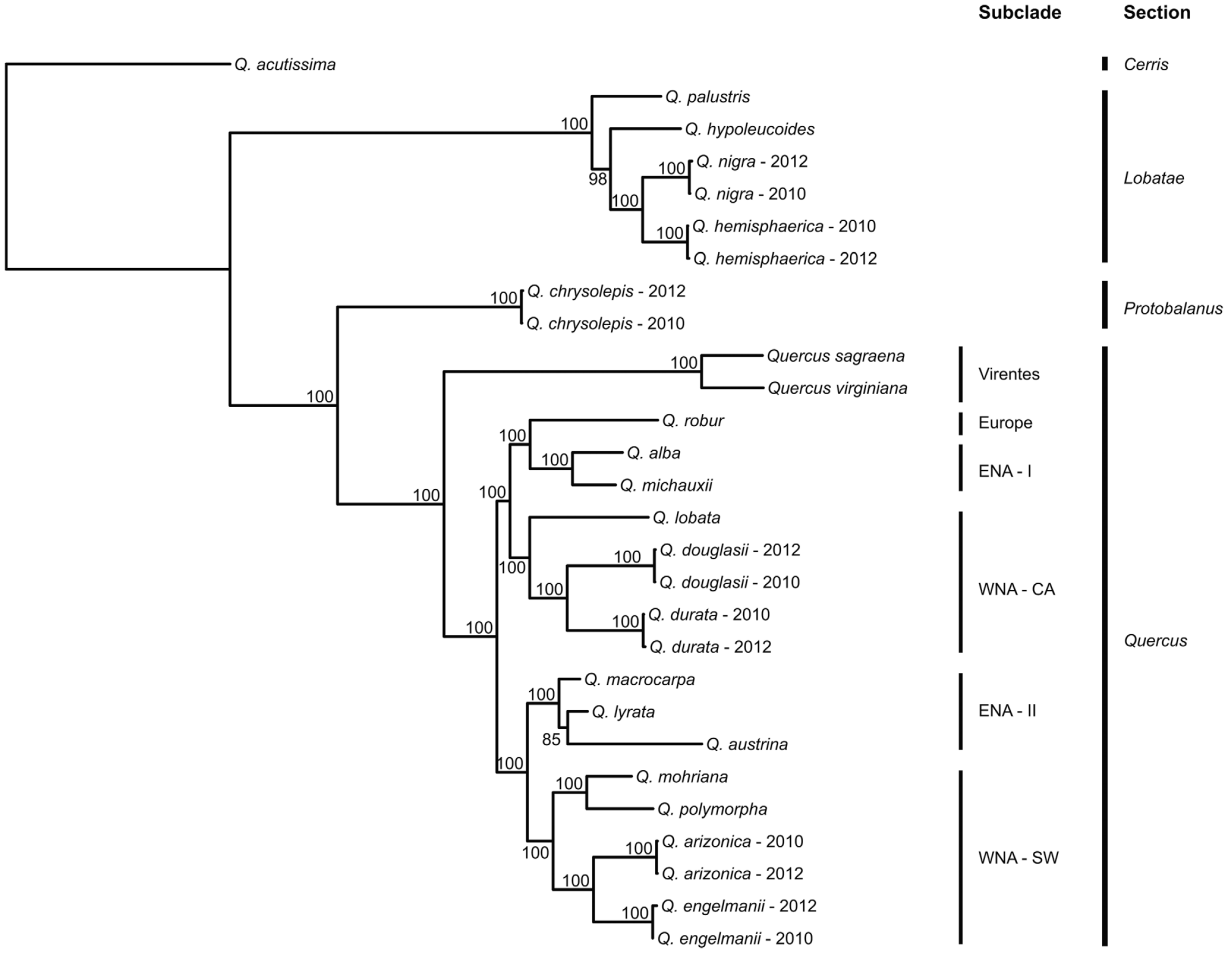
Ordination of *Quercus* samples based on nonmetric multidimensional scaling of locus presence-absence matrix. Spatial arrangement of points illustrates among-individual similarity in locus sampling. Red points represent sequences generated in 2010; technical replicates from the same library preparations are represented by black points, connected to their 2010 sequences by dashed lines.

**Figure 4:**
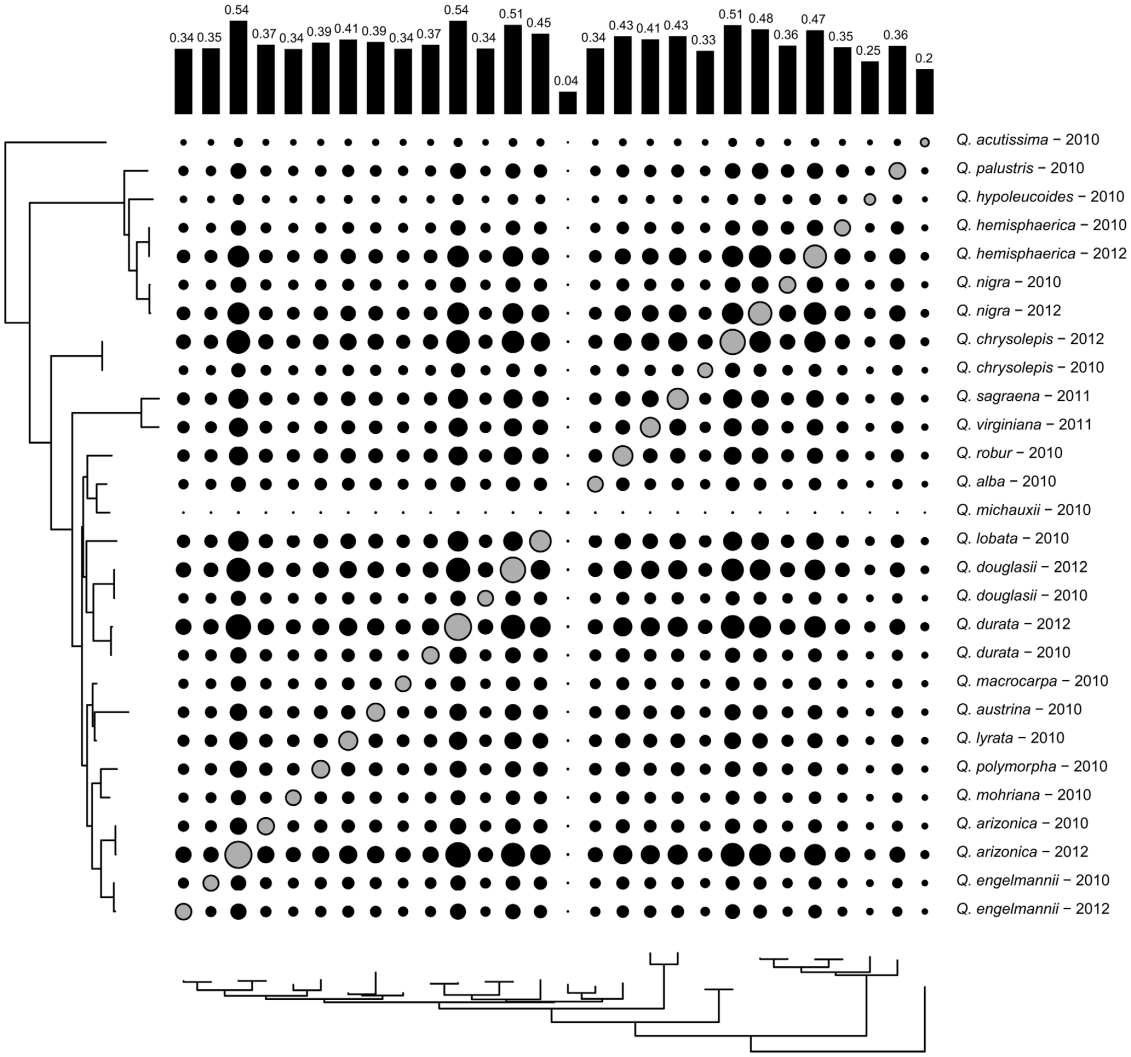
Proportion of loci shared among individuals. Loci shared between individuals (black circles, off-diagonal cells) or successfully amplifying within a single individual (grey circles along the diagonal) are expressed as the proportion from 0 to 1 of all 27,727 loci scored in this study. Bars at the top of the figure express the average percentage of loci shared by each individual, as an average of all the pairwise comparisons (black circles) for that individual. For scale, proportions range from a low of 0.022 (*Q. michauxii* – 2010 to *Q. acutissima* – 2010 [black]) to a maximum of 0.881(*Q. arizonica* – 2012 [gray]).

Ordination of the pairwise shared-locus matrix (where shared loci are scored as 1, loci not shared are scored as 0; Fig. 3) separates technical replicates, despite the fact that technical replicates fall next to each other with essentially no branch length separating them in the phylogeny (Fig. 2). While the pattern of locus-sharing is somewhat phylogenetically structured (Fig. 2, 4), the disparity between phylogenetic analysis of the underlying sequence data (Fig. 3) and ordination of the shared-locus matrix (Fig. 2) demonstrates that locus-sharing alone is not driving the phylogenetic signal recovered in this study.

Within section *Quercus* (the white oaks), we identify four main clades in addition to the Virentes group: two western North American clades, one composed of California species (WNA – CA) and one of southwestern North American and Mexican species (WNA – SW); and two predominantly eastern North American clades (ENA – I, ENA – II), one of which (ENA – I) is sister to the only Eurasian white oak sampled in this study (*Q. robur*). The western and eastern North American clades are interdigitated with strong support (100% bootstrap), and *Q. robur* is embedded within the American oaks with equally strong support. The phylogenetic relationships among sections matches prior work in the genus [24], [25], [30]. The division of the eastern North American white oaks (section *Quercus*) into two subclades is compatible with prior AFLP data [30], and the placement of *Q. palustris* as sister to the remainder of the *Lobatae* sampled is also compatible with prior allozyme work [80]. The placement of Eurasian white oaks (*Q. robur*) as sister to a subset of the eastern North American white oaks is novel to our study.

### Partitioned RAD analysis

**Figure 5:**
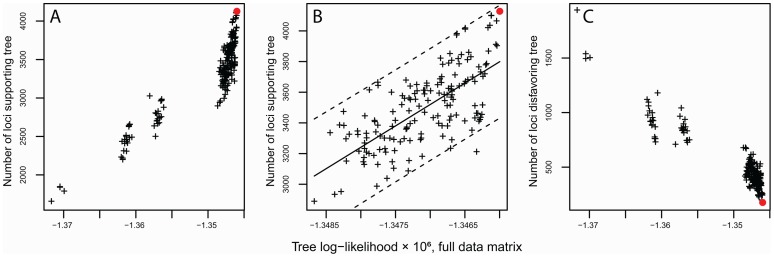
Partitioned RAD analysis: loci favoring or disfavoring globally suboptimal topologies. Each plus sign represents one of 200 phylogenetic trees in which the topology has been modified from the globally optimal tree using 1 or 2 steps of nearest neighbor interchange (NNI). The red filled circle indicates the globally optimal tree. Loci were included in this analysis only if the total range in log-likelihood across all trees was 4.0 or more and at least 20 unique phylogenies remained after pruning taxa not present in the locus. Each locus was counted as favoring trees that fell within 2 log-likelihood points of the best-supported tree for that locus, and disfavoring trees that fell within 2 log-likelihood points of the most poorly supported tree for that locus. **A**. All 201 trees, with y-axis indicating number of loci favoring each tree. **B**. Only trees supported at log-likelihood > -1.35E06, with y-axis indicating number of loci favoring each tree; dashed lines enclose the 95% prediction interval. **C.** All 201 trees, with y-axis indicating number of loci disfavoring each tree.

The globally optimal tree is strongly favored in our dataset, with a log-likelihood of ⋅1,345,997, compared to the next best tree with a log-likelihood of 1,346,038 (Figs. 5A, B); 4,128 supporting loci, compared to the next best tree with 4,093 supporting loci (Figs. 5A, B); and the smallest number of disfavoring loci (Fig. 5C). There is a strong disjuncture between topologies supported at log-likelihood > 1.350E06 and those < 1.355E06 (Fig. 5A). Topologies with the higher likelihood have the red oaks and the *Virentes* both supported as monophyletic; those in the lower likelihood groups break up the red oaks, *Virentes*, or both. Both of these clades are strongly supported by all previous studies [24], [25], [30], and hybridization appears not to occur between the white and red oaks [16], [18], suggesting that the more poorly supported islands of trees (<⋅1.355E06) are not plausible alternative topologies. Removing these poorly supported islands yields an island of trees that apparently draw from a single likelihood distribution (Fig. 5B). No topologies fall above/to the left of the 95% prediction interval on this distribution, suggesting that there is not a topology supported by a disproportionately large suite of loci, as we might expect from a relatively small number of hybridization events.

### Gene ontology annotation of RAD dataset

Of 27,727 loci in the m10 RAD dataset, 5,197 (18.7%) were inferred to correspond to protein coding regions in at least one of the expressed sequence databases queried using BLASTN at E-value < 9E–15, 4,715 (17.0%) at E-value < 1E–15. This suggests that the majority of the loci used in our RAD phylogeny fall into non-coding regions (e.g., promoter regions, introns and UTRs). BLASTN on the relatively small RefSeq RNA database returned 77 matches (i.e., 77 loci match at least one sequence at E-value < 9E–15). 2,024 loci BLAST to a sequence in the *Quercus* EST database, and 5,093 BLAST to a sequence in the ‘EST-others’ database; 1,936 loci BLAST to a sequence in both the *Quercus* EST database and the ‘EST-others’ database. 1,844 loci BLAST to only one expressed sequence, 1,027 to two, the remainder to more than two. Loci with multiple BLASTN hits often BLAST to sequences that appear in downstream GO annotation analysis to be similar to each other based on description. For example, locus 25,806 has a total of 736 BLASTN hits among the 3 databases, but all subject hits correspond to the light-harvesting complex.

At least one GO term was found to apply to 4,309 loci, and only 202 loci match to one and only one GO term. No GO term could be determined for 888 loci. The average number of GO annotations per locus is 12.78, while the maximum is 71. The rank order of GO term abundance is relatively insensitive to which database we utilize, and we consequently report here only on GO annotations for the 1936 loci with BLASTN hits to both *Quercus* EST and ‘EST-other’ (Figs. S1a, b). The most abundant GO terms are cellular processes (1269 loci), response to stress (933 loci), biosynthetic processes (930 loci) in the subcategory biological processes; protein binding (586 loci), nucleotide binding (357 loci), catalytic activity (334 loci) in the subcategory molecular function and plasma membrane (682 loci), cytosol (596 loci) and plastid (568 loci) in the subcategory cellular compartment (Figs. S1a, b).

### Phylogenetic informativeness of RAD loci associated with Expressed Sequence Tags (ESTs)

Putative homologs to coding regions exhibit a significant (*P*<0.01) 41.4% decrease in potentially parsimony-informative nucleotide positions relative to the number in a draw of the same number of loci from the remainder of the dataset (Table 1). However, this decrease from 9,515±175.1 to 5,574 potentially variable nucleotides still samples a substantial amount of data compared to the number of nodes needed to reconstruct this phylogeny. Missing data drop from 43.8% (±0.2%) in the non-EST dataset to 36.2% in the putative homologs dataset (*P*<0.01), and consistency index (CI) increases moderately but significantly (from 0.840±0.004 to 0.872, *P*<0.01; Table 1). Mean bootstrap drops slightly but non-significantly (from 0.940±0.022 to 0.912, *P* = 0.28; Table 1). The only taxon identified as phylogenetically unstable across replicates based on rogue-taxon analysis is *Quercus michauxii*, which had the lowest overall sequencing coverage of all samples sequenced (Figs. 1, 4). Relative branch length in the tips of the tree did not differ between the non-EST dataset and the dataset composed of putative homologs to coding regions (*P* = 0.48).

## Discussion

Reconstructing the oak tree of life has long been elusive. Our study demonstrates the utility of RAD data for reconstructing phylogenetic relationships in a problematic group that spans a 23–33 million-year-old divergence. It also demonstrates the feasibility of identifying genes underlying that phylogeny, information that we can use to investigate how gene function influences phylogenetic informativeness.

### Sequenced RAD markers for phylogenetic inference

The pattern of locus-sharing among individuals loosely reflects phylogenetic history, as evidenced by the fact that, for example, accessions of sections *Lobatae* and *Quercus* sequenced in 2010 cluster together in an ordination based on the locus presence-absence matrix (Fig. 3), and clustering within those sections (Fig. 3) largely follows the geographic subclades identified in the phylogeny (Fig. 2). However, the 2012 technical replicates do not cluster near their 2010 counterparts (Fig. 3), due to the relatively high coverage in the 2012 sequencing runs (Fig. 1) and the relatively small number of loci shared between individuals in each replicate pair (Table 1). Despite this fact, phylogenetic analysis of the sequence data, treating missing loci as missing characters, places all 2012 technical replicates sister to their 2010 counterparts, with terminal branches negligible in length (Fig. 2). This suggests that missing data have little or no effect on species placement on the tree, at least in our dataset, and that data are readily combined across sequencing runs (cf. [81], [82]). This is a substantial improvement over AFLP data, in which combining data across separate analyses is time-consuming, requiring rescoring of the entire data matrix, and often presents technical challenges. Moreover, RAD data provide a vast increase in the amount of data that can be readily generated for a non-model organism over what has been possible with previous-generation genotyping methods: the current study samples 1.40E06 aligned nucleotides, compared to the previously published AFLP phylogeny of *Quercus* [30], which sampled an estimated 4.7E04 bp in the recognition sites flanking the AFLP bands.

RAD data may capture multiple phylogenetic stories that are difficult to tease apart, because each locus is short and cannot support many nodes on its own. The partitioned RAD method we present here provides a means of exploring alternative topologies that may be supported by a large suite of loci but not readily identified using formal species-tree methods. Our analyses suggest that there is not a single tree or small number of trees that are supported by a disproportionately large number of loci, considering their likelihood. The framework oak phylogeny we present is thus not likely to be masking a lower-likelihood tree that nonetheless has a disproportionately large number of loci supporting it, as we might expect if this topology were dominated by hybridization between a few species. When we speak of the oak phylogeny, we can talk meaningfully about divergence history. What this analysis does not reflect is the history and direction of introgression in oaks. The partitioned RAD analysis presented here may complement more sensitive hypothesis-testing methods developed for genome-scale data (e.g., [50]), aimed at identifying specific introgression events.

### Association between RAD loci and expressed sequences

Because of the quality and volume of sequence data obtained using next-generation sequencing of the RAD library, use of sequenced RAD data provides a new opportunity to link phylogenetic study with research into genome function and structure. In the current study, we used BLAST searches of our consensus loci against expressed sequence databases to investigate (1) the genetic identity of the sequences we are using to estimate the oak phylogeny, and (2) the ability of EST-associated loci relative to non-EST-associated loci to resolve the framework phylogeny of oaks. We had expected that EST-associated loci would disproportionately resolve deeper nodes of the phylogeny, thus decreasing the relative length of branches subtending technical replicates (compared to the entire tree). In fact, we found no such trend (Table 2). We also expected that EST-associated loci would exhibit lower homoplasy, less missing data, and lower phylogenetic variance (higher bootstrap) than an equivalent number of non-EST-loci. While the first two predictions were true with strong support (*P*<0.01), the EST-associated loci exhibited reduced average bootstrap across the tree, though not significantly (*P* = 0.28). Based on rogue-taxon analysis [78], [79] and visual inspection of trees, the phylogenetic instability introduced appears to be due largely to movement of *Q. michauxii* and the two species most closely related to it (*Q. robur* and *Q. alba*). Low data coverage in *Q. michauxii* alone very likely accounts for this result: in 1000 random resamples of 5,197 loci, *Quercus alba* and *Q. robur* shared an average of 2,056.5 loci, while *Q. alba* and *Q. michauxii* shared an average of only 176.1. Moreover, *Q. michauxii* shares the lowest number of loci with other taxa of any taxa in our study, and has the lowest overall number of loci sequenced (Fig. 4).

**Table 2:** Phylogenetic statistics for loci that BLAST to one of three expressed sequence databases (‘EST’) relative to an equal-sized subsample of loci that do not (‘non-EST’) as well as the full m10wRE dataset (‘Full’).

	Full	EST	non-EST	*P*
**Steps**	87,668	8,674	16,420±183.3	<0.01
**Variable characters (number)**	112,565	12,871	20,643±170.2	<0.01
**Potentially parsimony-informative characters (number)**	51,378	5,574	9,512±104.4	<0.01
**Consistency index (CI)**	0.844	0.872	0.841±0.004	<0.01
**Aligned matrix length (nucleotide positions)**	1,397,722	233,669	234,575±43	<0.01
**Proportion missing data (N or -)**	0.424	0.362	0.439±0.002	<0.01
**Proportion of total branch length in the species tips**	0.168	0.180	0.177±0.004	0.48
**Mean bootstrap**	99.5	91.2	93.84±2.449	0.28

Statistics were calculated for the full m10wRE dataset, the dataset composed only of the 4,715 loci that blasted to at least one of three expressed sequence databases with E-value < 1E–15, and 100 randomly subsampled datasets of 4,715 loci drawn at random loci that did not blast at any level to the expressed sequence databases. For both the ‘EST’ dataset and the ‘non-EST’ subsamples, loci were drawn from the subsample of loci that were between 50 bp and 55 bp aligned length, inclusive, and statistics for each dataset are calculated on the maximum likelihood tree for that dataset. *P*-values approximate the type-I error rate under the null hypothesis that the ‘EST’ loci are drawn from the ‘non-EST’ pool of loci. *P-*values are calculated as two times the percent of random subsamples that are more extreme than the statistics observed on the ‘EST’ tree.

One of our goals in this study was to determine what gene functions are represented among loci we sampled in our estimate of the oak phylogeny. Since our RAD dataset represents sequences of coding as well as non-coding regions of the oak genome, finding GO terms for all loci was not expected. However, our work demonstrates that the markers we are using for phylogenetic inference are far from anonymous: 4,715 (17.0%) of the loci used in the phylogenetic study could be matched to an expressed sequence with relatively high certainty (E-value < 1E–15), and GO terms could be assigned to 4,309 of these target sequences. While our sampling of exemplars from across the American oak clade is poorly suited to relating phylogenetic information and patterns of allele-sharing among lineages to biogeography and selective regimes, many of the loci in our dataset represent genes involved in cellular processes, response to stress, protein binding and plasma membrane. This points to the potential to link RAD data to functional gene data, allowing us to mine these genotype data to address a wide range of questions in molecular evolution and adaption.

### Phylogeny and classification

The topology of the American oak clade has previously been hypothesized to be (*Lobatae*, (*Quercus* s.s., *Protobalanus*)), but not with strong support from robust DNA sequence data [24], [29], [30]. Our finding that this topology is well supported, combined with the placement of the Virentes clade sister to the remainder of the white oaks, provides the framework needed for further study of the clade.

Despite our very sparse sampling, two additional phylogenetic results stand out in this study within section *Quercus* (the white oaks): the separation into small geographic clades, a result also shown in the previous AFLP study [30], with the eastern North American taxa non-monophyletic; and the possible placement of the Eurasian members of section *Quercus* (the roburoids) within or sister to one of the eastern North American clades. In contrast, prior AFLP data placed the roburoids sister to the non-*Virentes* members of section *Quercus* from North America [30], and a study utilizing nuclear ribosomal DNA sequences had suggested a relationship between the western North American *Q. sadleriana* and *Q. pontica* of the western Caucasus Mountains [29]. Additional sampling will be needed to test whether there are in fact two intercontinental disjunctions within section *Quercus*. In the meantime, these findings lay the groundwork for a meaningful subsectional classification of *Quercus* based on phylogeny, geography and morphology.

## Supporting Information

Figure S1 a, b. GO term distribution by database. Pie charts of the 1936 loci that had BLASTN hits in both the ‘*Quercus* EST’ and ‘EST-others’ databases, and GO term distribution for each category. Cellular components are represented in blue, molecular functions are represented in purple and biological processes are represented in green. Only the top 15 GO terms in terms of locus count are reported for each GO category (cellular compartment, molecular function, and biological processes).
(ZIP)
