# Correction: A Framework Phylogeny of the American Oak Clade Based on Sequenced RAD Data

**DOI:** 10.1371/journal.pone.0102272

**Published:** 2014-07-02

**Authors:** The PLOS ONE Staff

The images for Figures 2 and 3 are incorrectly switched. The image that appears as Figure 2 should be Figure 3, and the image that appears as Figure 3 should be Figure 2. The figure legends appear in the correct order.
