# Supplementary figures and images for: A Framework Phylogeny of the American Oak Clade Based on Sequenced RAD Data
Source: PLoS One. 2014 Apr 4;9(4):e93975. doi: 10.1371/journal.pone.0093975 (PMC3976371; doi:10.1371/journal.pone.0093975)

1936 loci GO annotation represented in *Quercus* EST+ EST others

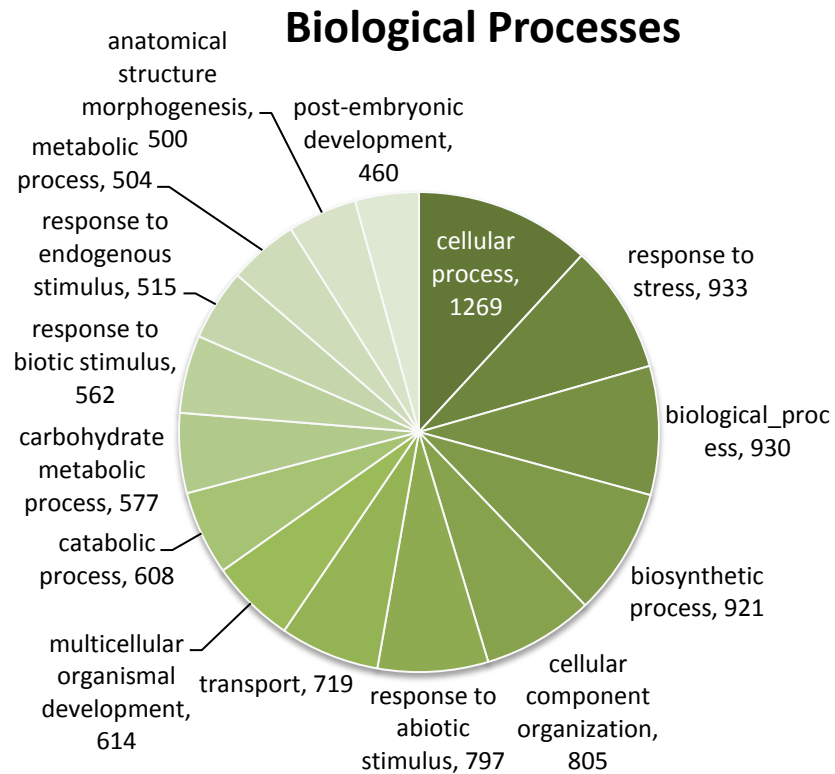

Supplement: Figure S1 — a, b. GO term distribution by database. Pie charts of the 1936 loci that had BLASTN hits in both the ‘Quercus EST’ and ‘EST-others’ databases, and GO term distribution for each category. Cellular components are represented in blue, molecular functions are represented in purple and biological processes are represented in green. Only the top 15 GO terms in terms of locus count are reported for each GO category (cellular compartment, molecular function, and biological processes). (ZIP) [file pone.0093975.s001.zip › HIPP.oaks.FIGS1b.GOs.biol_new.pdf]
